# An integral approach to address Chagas disease

**DOI:** 10.3389/fpara.2023.1114563

**Published:** 2023-04-05

**Authors:** Marina Gold, Maria Julia Hermida

**Affiliations:** ^1^Fundación Mundo Sano, Buenos Aires, Argentina; ^2^Institut für Sozialanthropologie und Empirische Kulturewissenschaft (ISEK), Universidad de Zurich, Zurich, Switzerland; ^3^Instituto de Educación, Universidad Nacional de Hurlingham, Villa Tesei, Provincia de Buenos Aires, Argentina; ^4^Consejo Nacional de Investigaciones Científicas y Técnicas, Buenos Aires, Argentina

**Keywords:** Chagas, primary health care, integrated approach, community engagement, diagnosis and treatment, social science & humanities

## Abstract

Chagas is a zoonotic disease conditioned by the need to eliminate or control the vector in human settlements before targeting infected individuals. Simultaneously it is necessary to raise awareness of health problems generated by chronic Chagas disease (ChD), for people to participate actively in vector control programs that will then enable the implementation of screening, treatment and follow-up strategies. Therefore, it is essential to engage the participation of the community in holistically designed integral programs to address ChD in all its complexity. This Perspective presents the case of Chagas management programs in the Department of General Taboada, Province of Santiago del Estero, Argentina, to showcase a possible strategy in vector control, diagnosis and treatment programs that integrate ChD into the local public health system and engage community participation. Through this Perspective we argue for the importance of the contribution of social science methodologies and epistemologies in the process of integrating ChD into the public (and primary) health care system.

## 1 Introduction: Social determinants of health in Chagas disease

Health is a complex phenomenon extending beyond disciplinary silos (Albrecht et al., 1998). To address the complexity of health issues, the World Health Organization introduced the concept of Social Determinants of Health (SDH), defined as “non-medical factors that influence health outcomes” and include a wide range of social and systemic factors related to conditions in which populations or individuals are born, grow, live, work, and age (World Health Organization, 2022). Social determinants of health are especially informative in the case of neglected diseases of poverty, such as Chagas disease (ChD) and they need to be considered in the implementation of prevention, control, and treatment strategies in endemic and non-endemic areas.

Poverty is the main SDH in ChD (Briceño-León and Méndez Galván, 2007; Gürtler, 2009), although, as a product of migration ChD is also currently endemic in economically affluent regions (Hotez, 2018). Precarious living conditions associated with poverty (e.g., mud housing, cohabitation with domestic animals, insufficient food hygiene practices, lack of running water), are linked with a higher incidence of ChD (Ventura-Garcia et al., 2013; Fernández et al., 2019).

Social determinants of health increase the value of interdisciplinarity in addressing ChD, a point some scholars have argued for decades (Rifkin, 1996; Ventura-Garcia et al., 2013). The precursors of approaches that aim to include SDH first emerged within the purely medical sphere (Briceño-León, 2003). Subsequently, through the increasing influence of social sciences, SDH (from the individual level to collective practices) began to be considered among the main causes of some diseases (Azoh Barry, 2014). However, although recent work has shown that SDH are gaining ground (Reidpath et al., 2011), much of the research on ChD categorized as “social science” or as “considering SDH,” upon closer inspection tends to be carried out in support of the implementation of biomedical approaches (Reidpath et al., 2011). Additionally, social science programs on neglected tropical diseases (NTDs) receive significantly less funding (35%) than those classified as hard sciences (Pokhrel et al., 2011). In practice, programs often lack a robust socio-cultural perspective that considers SDH from their inception, making them only partially effective.

In this perspective we present a program that has considered SDH from its beginnings, contemplating the problematic of ChD from its multiple angles and projecting a sustained and transversal strategy. In this program, SDH are at the core of its design and execution. Through the description of a case study of implementation of ChD management in the Department of General Taboada, Santiago del Estero (Argentina) we aim to showcase a possible strategy of an integral inclusion of SDH into vector control, diagnosis, and treatment programs aiming to interrupt the transmission cycle of Trypanosoma Cruzi parasite to humans.

## 2 The benefits of including SDH at the core of a ChD control program: The case of General Taboada, Santiago del Estero, Argentina

**Figure 1:**
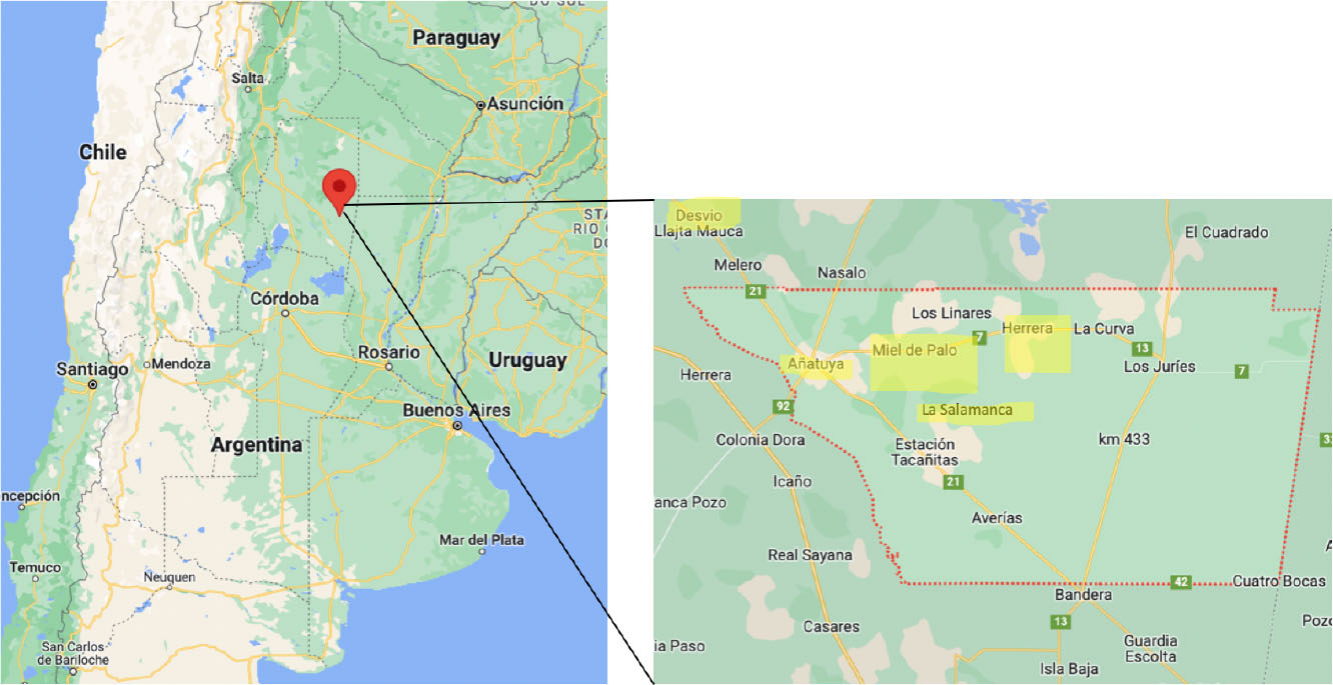
*Intervened locations.* Argentina, Santiago del Estero, General Taboada.

The province of Santiago del Estero, located in the northwest of Argentina (**Figure 1**), has 1.054.028 inhabitants (INDEC, 2023), and the highest prevalence of ChD in the country, with more than 200,000 people affected by *Trypanosoma Cruzi* (Silveira et al., 2002). The presence of the vector is only one conditioning factor, with socio-environmental elements increasing the risk of infection: such as the characteristics of the building material of traditional dwellings (constructions with adobe walls, rammed earth floors and roofs of mud, straw and sticks), the practices of animal husbandry that lead to cohabitation and close contact with animals, who also move through the adjacent environment coming into contact with the vector, and the lack of awareness of possible treatment and prevention strategies.

Previous control strategies practiced by local authorities have mostly focused on intra- and peri-domiciliary spraying with residual insecticides, which have not resulted in the interruption of vector-borne transmission, and high infestation rates of *Triatoma Infestans* persist (Abril et al., 2009). Most vector control programs disregard the importance of considering socio-environmental factors: the constant interaction between people, animals and vectors given the living patterns of local inhabitants. The absence of community participation in traditional vector-control programs has missed the opportunity to engage the community in a process of empowerment through education and the transfer of skills, seeing them as passive recipients. Yet, community participation is an important factor in the success and sustainability of vector control programs (Bryan et al., 1994; Abad-Franch et al., 2011).

Taking these factors as a starting point, in 2001 the Argentinean non-government organization Mundo Sano started working in diagnosis and treatment programs in the city of Añatuya, which had been exposed to national programs of vector control and surveillance. The city of Añatuya is located two hundred kilometres southeast of the capital of Santiago del Estero. It is the only urban centre in the Department of General Taboada and has more than 20,000 inhabitants. In 2006, after long-standing presence in the region, a holistic program for the control, diagnosis and treatment of ChD in rural areas of the department of General Taboada was designed and implemented by Mundo Sano, considering socio-cultural factors and focusing on community engagement. The project consisted of subsequent intervention phases that ranged from entomological surveillance and control, georeferencing of dwellings, different stages of home improvement and dissemination of education material, activities through schools and community events and the establishment of Chagas specialists in the local health centres (see **Table 1** for a description of the size of the program). The initial driver behind the project was to interrupt vector control in rural areas in order to treat people without the risk of reinfection. Therefore, from its conception, the project had a *long durée* vision, understanding that the problematic of Chagas has no quick and simple solution. The following factors were key for its success:

**Table 1:** Number of houses, inhabitants and percentage of improved houses by intervened location.

Site	Number of houses	Number of inhabitants	% of improved houses
Desvío	53	201	83.7
Miel de Palo	81	314	96.4
Herrera	39	103	78.4
La Salamanca	36	138	76.4

Data obtained until 2019. More information available in Weinberg et al., 2019 and Weinberg et al., 2018.

### 2.1 Community participation

The integral Chagas control program in General Taboada was designed and carried out in a consultative process, in constant collaboration with local and provincial government bodies and other relevant stakeholders to guarantee its acceptance and sustainability (Weinberg et al., 2018). Simultaneously, through initial information sessions, the team at Mundo Sano identified the relevant community leaders that could recruit and help coordinate each home improvement intervention. Other local institutions were present at initial information sessions, such as school staff, local health care center staff and local religious institutions, three key community structures.

Additionally, Mundo Sano engaged anthropological experts at different points in the development of the project to conduct socio-cultural situational analysis to understand people’s social networks, their relationship to land, and conceptions of living space and livelihoods. This enabled the team at Mundo Sano to comprehend the importance of the socio-cultural context of the population in question, and encouraged respect of local practices, such as maintaining the style of dwelling and the materials used, which keep houses cool in the high summer temperatures (Goldberg, 2008; Mastrangelo, 2009). Furthermore, it reinforced the importance of considering SDH in the design and implementation of Chagas-related programs, as most respondents associated their plight with Chagas with their lack of resources to construct a house made of bricks (Goldberg, 2008; Kaul et al., 2018). Such anthropological studies are fundamental in understanding how messages are received and how best to direct them within the target community. For example, in the education process of reorganization of the house and peri-domicile area to reduce vector presence, it was women who had taken in the message more consciously than men, as animal husbandry and household work was their domain (Goldberg, 2008). The inclusion of the anthropological perspective might have been a key factor for the results. With intradomiciliary infestation average was reduced by 85%: initially 40% of households showed domiciliary infestation, while only 5% proved positive for Triatomines after interventions (Fundación Mundo Sano, 2021).

The starting point of every intervention was a community meeting at the location to be intervened, where all interested groups were identified, and the program explained. The working groups were configured by the local team of Mundo Sano, identifying community leaders, aiming to build coherent working groups by living proximity or kin. Throughout the work process in each house the team at Mundo Sano was present to ensure continuous capacity building support, as well as conflict management wherever it arose. While Mundo Sano donated building material and provided technical support through the intervention of a construction officer, a key element of the program required that all locations established their working groups that would carry out the work in every house, assisting those households who had no male members to carry out the construction work. The purpose of this practice was to empower local inhabitants in building techniques that they would need to periodically employ to fix up their houses. Proof of the success of this empowering process is the replication of building techniques by households that were not part of the program but appreciated the benefits of the waterproof roof and the whitewashed walls. These households received support from those who had learnt the procedures from the construction officer.

Furthermore, this methodology considered SDH such as labor capacity and gender roles. Houses where male members were either unable to carry out construction work due to disability or age, or were absent due to the need to work in seasonal harvest, were supported by the working team of neighbors.

**Figure 2:**
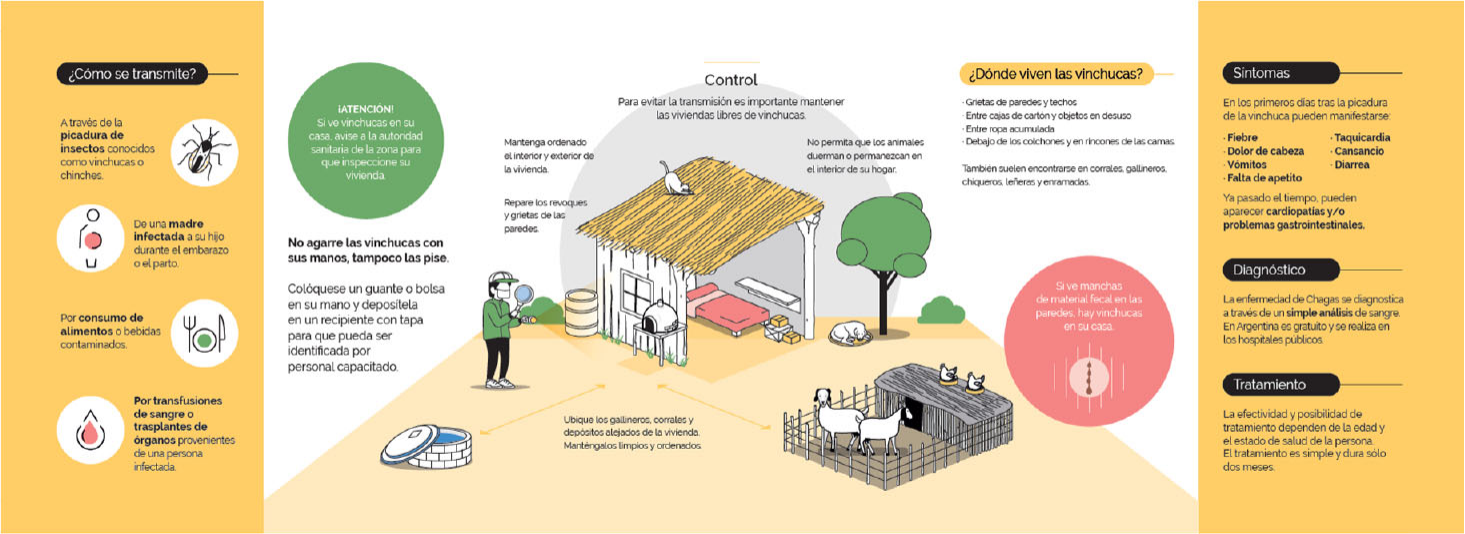
Part of a booklet used in the program (in Spanish, the local official language of the community). The booklet briefly describes vector transmission, symptoms, diagnosis and treatment, and highlight (figure in the center) the actions needed to control the vector intra and pero domicialiary.

Once the groups were formed, they first received theoretical and practical training on building techniques, water management sanitation, management of human waste, reorganization of the peridomicile area with the relocalization of animals in enclosures and chicken coups, providing vector surveillance and control techniques. In parallel, information sessions are organized in schools about ChD transmission and hygienic practices to avoid contact with the vector, in order to multiply the points of dispersion of information and to engage relevant community institutions in the integral approach to the issue (**Figure 2** shows an example of the educational material used in the program). This shows that education (another SDH) was included in this program.

### 2.2 Adaptive methodology to the needs of the participants

One of the greatest strengths of the project, which has enabled it to so aptly contemplate the importance of SDH, has been the capacity to adapt its methodology and procedures, taking into consideration needs and risks as perceived by local inhabitants. This is also a component of participative methodologies, which constantly reinforce feedback mechanisms and enable adaptive strategies to contemplate unforeseen local needs.

**Figure 3:**
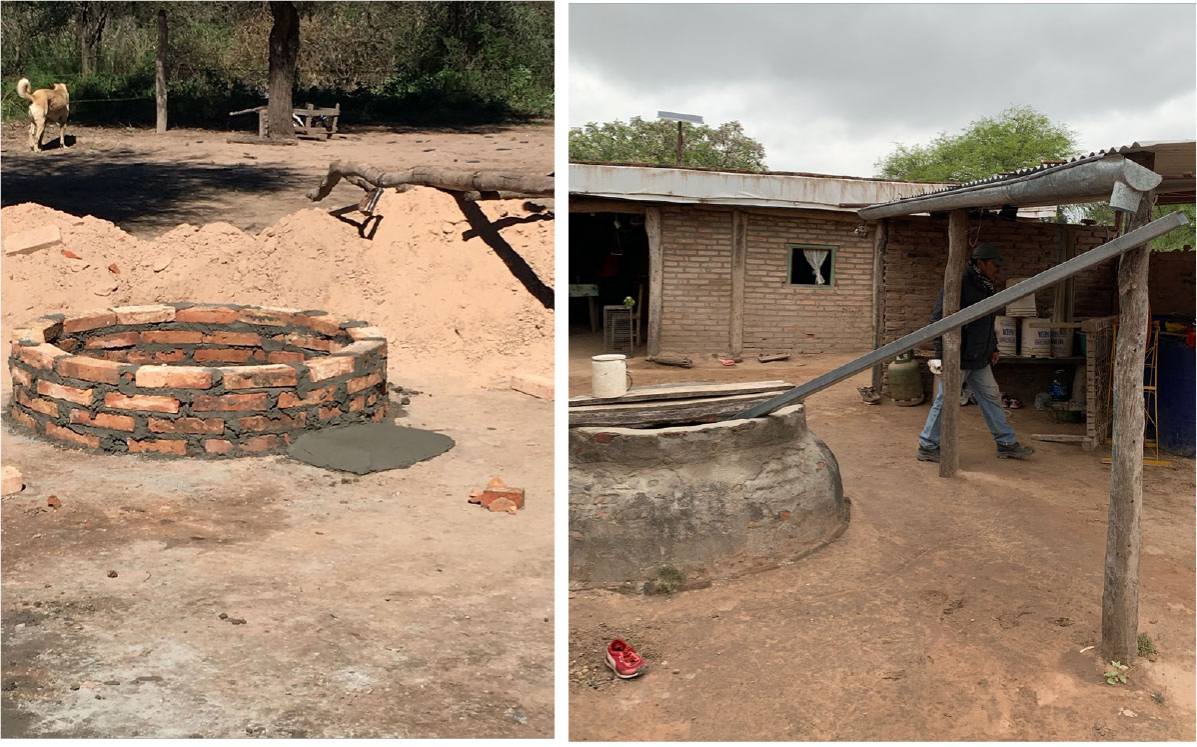
Water well examples.

**Figure 4:**
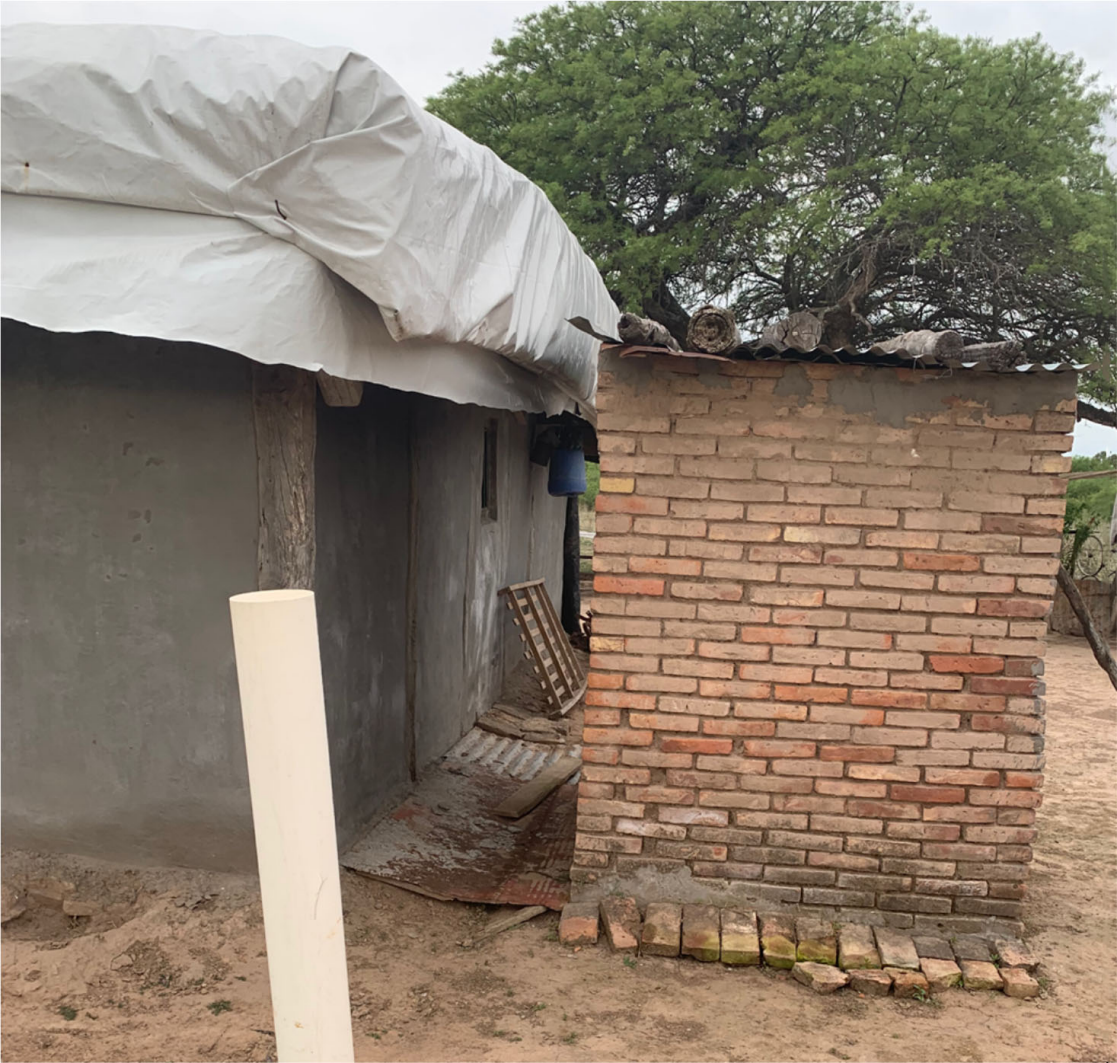
Outdoor latrine.

**Figure 5:**
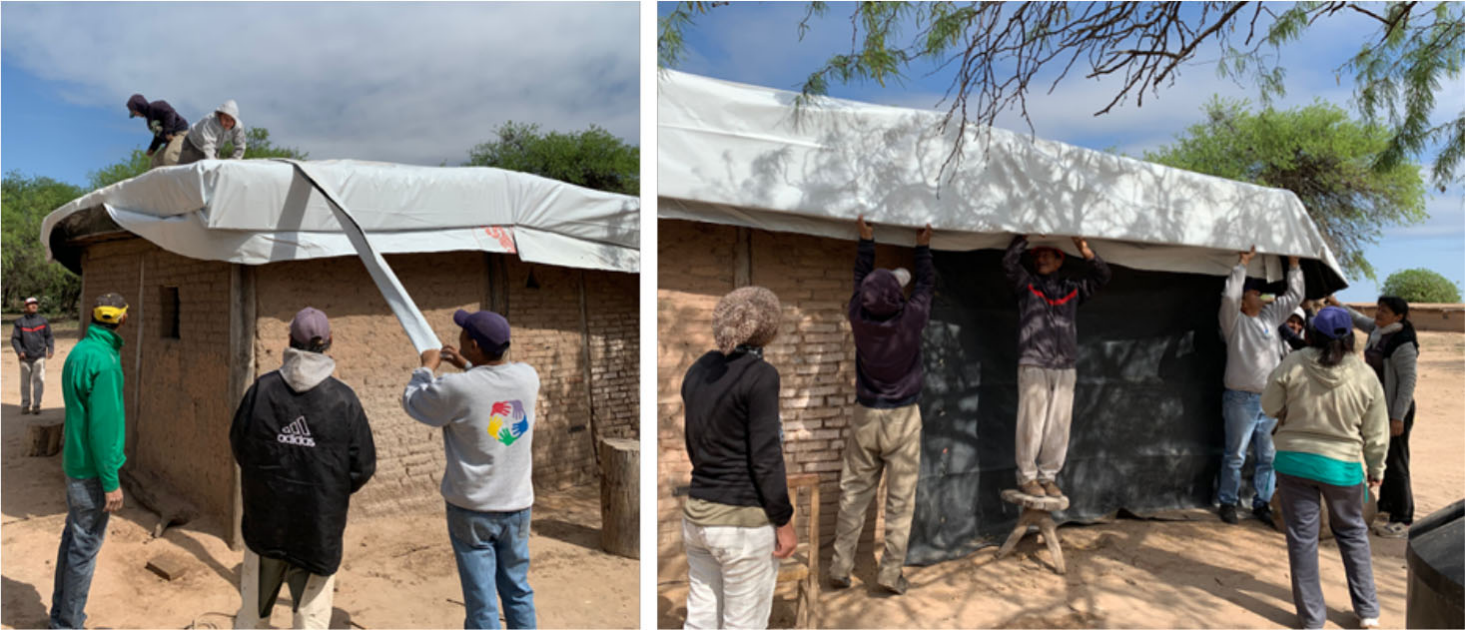
*Community working groups.* Pictures show community members working in groups on the improvement and waterproofing of roofs. Members worked on all houses in the location, although they do not lived in those houses, showing it was a communitarian and individual work.

**Figure 6:**
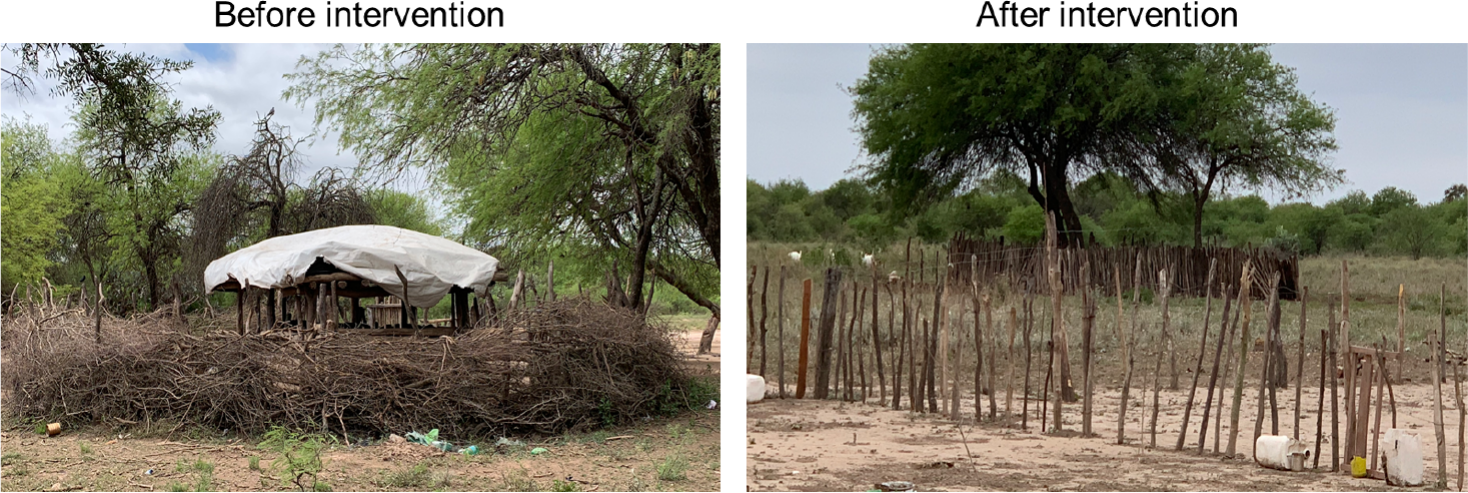
Animal pen examples.

The current intervention procedure per household – a direct result of integrating the community’s requests – is the following:

1. Construction of a well for water storage (capacity of 3,000 liters) (**Figure 3**);
2. Construction of an outhouse with a toilet and sink for hand washing (**Figure 4**);
3. Construction of a space inside the home for adequate handling and processing of food;
4. Plastering and whitewashing of all inside walls;
5. Improvement and waterproofing of all roofs (**Figure 5**);
6. Plastering and painting of all external walls;
7. Household and peri-domicile cleaning and environmental management and construction of animal pens (**Figure 6**). These are relocated if needed so that they are at least 50 meters from the household. Both of these activities are transversal to the rest of the improvements; they begin with the water well construction and continue as long as the program is in progress (Abril et al., 2009).

The initial construction of the water well (**Figure 2**), a cornerstone of the intervention as it is the most desired element of the improvement process by local inhabitants given the scarcity of water, is a direct response to demands and interests of the local community. It was this deliverable that made the home improvement project appealing to many hamlets, which may have otherwise not engaged in the project so willingly. The other element provided by the project, which is not directly connected to the prevention of ChD, has been the latrine (**Figure 3**), further encouraging people to participate in the home improvement project, and granting it a further layer of complexity by generating a more hygienic peri-domicile.

The use of local materials (mud and local grasses), and the respect of local building practices (Kaul et al., 2018) has also resulted in the positive reception of the programs, as people were not imposed a foreign structure that was not appropriate to local cultural living practices. In contrast, the provincial program for the eradication of mud dwellings (viviendas rancho) aimed to replace the rancho with brick houses, and utilized metal slats as roofing, making them uninhabitable in the high summer temperatures.

Since the program started in 2005, 495 houses were improved benefiting 1822 people, more than 400 waterholes were built, more than 400 latrines were installed, and 300 capacity building sessions were carried out.

Once the home improvement and vector control process had been sustainably ongoing long enough to generate a decrease in transmission (Weinberg et al., 2018), Mundo Sano complemented the project with strategies to strengthen the diagnosis and treatment of Chagas in primary health care units at the local health care centers by providing medical staff with the appropriate training to diagnose, treat and follow ChD. This completed the integral nature of the program, by addressing it from its multiple angles: vector control, home improvement, capacity building and education of local communities, diagnosis, access to treatment and follow-up procedures. For all this infrastructure to be maintained, it was essential to articulate positive and committed relationships with local institutions and authorities.

### 2.3 Working jointly with local authorities

The sustainability of the project relies on its interlacing with local authorities and community institutions, such as schools, local health centers and local municipal leaders. Central to the sustainability of the intervention has been the alignment with the provincial program for Chagas control, and a legal agreement with the provincial government, which enabled the presence of a representative of Mundo Sano within the Añatuya provincial hospital to support screening and treatment activities. Information sessions about locally appropriate strategies for vector control and food hygiene (Kaul et al., 2018) were organized in local schools helping to raise awareness of the multiple forms of addressing ChD, while the integration of ChD specialists in the local clinics sensitized the health system to properly receive patients and provide treatment. Furthermore, the participation of different local actors ensured the repetition of the key messages around Chagas, in order to subvert its neglected status. Dr. Elsa Segura, in collaboration with Mundo Sano and the non-government organization Caritas, has carried out research on the importance of the interaction between different social and communal institutions in the promotion of Chagas disease prevention and surveillance, revealing the centrality of social networks in addressing the issue in Añatuya, Santiago del Estero (Segura, 2005). The sustained community engagement is only possible if a long-term vision is present within the project, and if the programs are not subjected to the whims of political funding. While the ultimate objective was to treat people with ChD, it was first necessary to address vectorial transmission in order to reduce the risk of reinfection. This can only be done when an organization is committing its sustained presence in a region and has a long-term objective.

## 3 Discussion

Although it has been largely claimed that SDH should be included in ChD control programs, they are usually given a subsidiary place as most interventions are predominantly biological or medical in focus. Through practical examples, the aim of this perspective has been to demonstrate how SDH can be incorporated into programs and greatly benefit the program’s impact.

This case study showed the inclusion of SDH around three main axes: community participation, adaptive methodology to the needs of the participants and working jointly with official authorities. As opposed to previous control programs applied in Añatuya, which used mostly chemical approaches for vector control (i.e. insecticides), this program incorporated these three axes which highlight SDH as key factors in Chagas management programs. The central consideration of SDH implied that:

-All activities of the program were contemplated with and for the community as part of the design and application of the program. For instance, local inhabitants formed working groups for the construction activities.
-Activities were developed taking into account local social and cultural practices. For example, gender roles in the community were considered for planning and conducting construction activities.
-Activities were carried out in collaboration with local authorities, which gave the program continuation and acceptance. Community leaders, school staff, local health care center staff and local religious institutions and local inhabitants took part in the intervention actions.
-Activities were organized through social networks (not at individual but social level), which also increased engagement in the program. For example, constructions and educational activities were made in groups.

This is an example of a vector control program in which SDH were central from design to development. We consider the success of the program (a reduction of domiciliary infestation from 40% to 5%) is due, in part, to this inclusion of SDH. This project is ongoing as continuously carries out home improvement procedures to willing inhabitants.

## Data availability statement

The original contributions presented in the study are included in the article/supplementary material. Further inquiries can be directed to the corresponding author.

## Ethics statement

Written informed consent was obtained from the individual(s) for the publication of any identifiable images or data included in this article.

## Author contributions

MG and MH contributed equally to this work, both contributed with intellectual content, writing and critically revising the work. All authors contributed to the article and approved the submitted version.

## References

[B1] Abad-Franch F. Vega M. C. Rolón M. S. Santos W. S. Rojas de Arias A. (2011). Community participation in chagas disease vector surveillance: Systematic review. PloS Negl. Trop. Dis. 5 (6), e1207. doi: 10.1371/journal.pntd.0001207 21713022 PMC3119642

[B2] Abril M. Coto H. Weinberg D. Coppede M. E. Cejas R. G. (2009). Mejoramiento de viviendas con participación comunitaria para la prevención y el control de la enfermedad de chagas en comunidades rurales del sureste de Santiago del estero, república Argentina. Emf Emerg. 11 (1), 28–33.

[B3] Albrecht G. Freeman S. Higginbotham N. (1998). Complexity and human health: The case for a transdisciplinary paradigm. Cult Med. Psychiatry 22 (1), 55–92. doi: 10.1023/a:1005328821675 9657059

[B4] Azoh Barry J. (2014). Social sciences research on infectious diseases of poverty: Too little and too late? PloS Negl. Trop. Dis. 8 (6), e2803. doi: 10.1371/journal.pntd.0002803 24921244 PMC4055465

[B5] Briceño-León R. (2003). Las ciencias sociales y la salud: Un diverso y mutante campo teórico. Cien Saude Colet 8 (1), 33–45. doi: 10.1590/S1413-81232003000100004

[B6] Briceño-León R. Méndez Galván J. (2007). The social determinants of chagas disease and the transformations of Latin America. Mem Inst Oswaldo Cruz 102 (1), 109–112. doi: 10.1590/S0074-02762007005000095 17891277

[B7] Bryan R. T. Balderrama E. Tonn R. J. Pinto Dias J. C. P. (1994). Community participation in vector control: Lessons from chagas disease. Am. J. Trop. Med. Hyg. 50 (6), 61–71. doi: 10.4269/ajtmh.1994.50.61 8024086

[B8] Fernández M. D. Gaspe M. S. Gürtler R. E. (2019). Inequalities in the social determinants of health and chagas disease transmission risk in indigenous and creole households in the Argentine chaco. Parasitol. Vectors 12 (1), 1–18. doi: 10.1186/s13071-019-3444-5 PMC6487000 31029147

[B9] Fundación Mundo Sano (2021) Construir salud con la comunidad. Available at: https://www.youtube.com/watch?v=vkVsJwlP4s0.

[B10] Goldberg L. (2008). Análisis cualitativo de las representaciones sociales ligadas al proyecto: “Desarrollo, transferencia y evaluación del impacto de un sistema de prevención y control del mal de chagas y otras enfermedades relacionadas con condiciones ambientales desfavorables en comunidades rurales del sureste santiagueño. Reporte preparado para la Fundación Mundo Sano.

[B11] Gürtler R. E. (2009). Sustainability of vector control strategies in the gran chaco region: Current challenges and possible approaches. Mem Inst Oswaldo Cruz 104 (1), 52–59. doi: 10.1590/s0074-02762009000900009 19753458 PMC3072747

[B12] Hotez P. J. (2018). The rise of neglected tropical diseases in the “new texas”. PloS Negl. Trop. Dis. 12 (1), 1–15. doi: 10.1371/journal.pntd.0005581 PMC5773009 29346369

[B13] INDEC (2023). Censo nacional de población, hogares y viviendas 2022 : resultados provisionales. 1a ed (Ciudad Autónoma de Buenos Aires: Instituto Nacional de Estadística y Censos). Available at: https://censo.gob.ar/wp-content/uploads/2023/02/cnphv2022_resultados_provisionales.pdf.

[B14] Kaul M. A. Berdiñas M. P. Petrillo J. F. (2018) Estudio sociocultural. anexo I resultados de los índices. Available at: https://www.mundosano.org/wp-content/uploads/2022/11/Informe-Final.pdf.

[B15] Mastrangelo A. (2009) El Chagas según santiago. relaciones sociales, ambiente y enfermedad de chagas en un paraje de Santiago del estero, Argentina. Available at: https://www.conicet.gov.ar/new_scp/detalle.php?keywords=&id=29746&congresos=yes&detalles=yes&congr_id=1232710 (Accessed 8th October 2012).

[B16] Pokhrel S. Reidpath D. Allotey P. (2011). Social sciences research in neglected tropical diseases 3: Investment in social science research in neglected diseases of poverty: A case study of bill and Melinda gates foundation. Health Res. Policy Syst. 9 (2), 1–6. doi: 10.1186/1478-4505-9-2 21210999 PMC3022559

[B17] Reidpath D. D. Allotey P. Pokhrel S. (2011). Social sciences research in neglected tropical diseases 2: A bibliographic analysis. Health Res. Policy Syst. 9 (1), 1–12. doi: 10.1186/1478-4505-9-1 21210997 PMC3024304

[B18] Rifkin S. B. (1996). Paradigms lost: Toward a new understanding of community participation in health programmes. Acta Trop. 61 (2), 79–92. doi: 10.1016/0001-706x(95)00105-n 8740887

[B19] Segura E. (2005). Redes sociales para la vigilancia de la transmisión del trypanosoma cruzi (Chagas). ministerio de salud y medio ambiente. comisión nacional de programas de investigación sanitaria Vol. 2002 (CONAPRIS: BECA “RAMÓN CARRILO-ARTURO OÑATIVIA”).

[B20] Silveira A. C. Rojas de Arias A. Segura E. Guillen Vargas G. E. Russomano G. Chenone H.. (2002). El Control de la enfermedad de chagas en los países del cono sur de américa. historia de una iniciativa internacional 1991/2001 (Washington DC: OPAS).

[B21] Ventura-Garcia L. Roura M. Pell C. Posada E. Gascón J. Aldasoro E.. (2013). Socio-cultural aspects of chagas disease: A systematic review of qualitative research. PloS Negl. Trop. Dis. 7 (9), e2410. doi: 10.1371/journal.pntd.0002410 24069473 PMC3772024

[B22] Weinberg D. Lanfri M. Scavuzzo C. M. Abril M. Lanfri (2019). Evaluation and planning of chagas control activities using geospatial tools. Geospat Health 14 (2), 229–238. doi: 10.4081/gh.2019.786 31724372

[B23] Weinberg D. Porcasi X. Lanfri S. Abril M. Scavuzzo C. M. (2018). Spatial analyzes of triatomine infestation indices and their association to the actions of a chagas disease program and environmental variables during a 5-year intervention period. Acta Trop. 188, 41–49. doi: 10.1016/j.actatropica.2018.08.025 30142310

[B24] World Health Organization (2022) Social determinants of health. Available at: https://www.who.int/health-topics/social-determinants-of-health#tab=tab_1.

